# Anti-PEG IgM Response against PEGylated Liposomes in Mice and Rats

**DOI:** 10.3390/pharmaceutics3010001

**Published:** 2010-12-27

**Authors:** Masako Ichihara, Taro Shimizu, Ami Imoto, Yuki Hashiguchi, Yumi Uehara, Tatsuhiro Ishida, Hiroshi Kiwada

**Affiliations:** Department of Pharmacokinetics and Biopharmaceutics, Subdivision of Biopharmaceutical Science, Institute of Health Biosciences, The University of Tokushima, 1-78-1, Sho-machi, Tokushima 770-8505, Japan

**Keywords:** Polyethyleneglycol (PEG), PEGylated liposome, Anti-PEG IgM, Accelerated blood clearance (ABC) phenomenon, spleen

## Abstract

We have reported that PEGylated liposomes lose their long-circulating properties when they are administered repeatedly at certain intervals to the same animal. This unexpected phenomenon is referred to as the accelerated blood clearance (ABC) phenomenon. We recently showed that the ABC phenomenon is triggered via the abundant secretion of anti-PEG IgM in response to the first dose of PEGylated liposomes. However, the details of the underlying mechanism for the induction of anti-PEG IgM production are yet to be elucidated. The present study demonstrated that the spleen is a major organ involved in the secretion of anti-PEG IgM in mice and rats. Anti-PEG IgM production was detected in nude, T-cell deficient mice, but not in SCID mice with B- and T-cell deficiencies. These observations indicate that splenic B-cells secret anti-PEG IgM without help from T-cells. Sequential injections of PEGylated liposomes into the same mice did not promote isotype switching from IgM to IgG. Accordingly, PEGylated liposomes may function as a type-2, T-cell-independent antigen (TI-2 antigen) during anti-PEG IgM production. Although the underlying mechanism that causes an anti-PEG IgM response against PEGylated liposomes is not yet clear, our findings give implications in revealing the anti-PEG IgM response against PEGylated liposome.

## 1. Introduction

It is well known that surface modification of liposomes with polyethylene glycol (PEG) improves the pharmacokinetics of liposomes after intravenous injection. The hydrophilic polymer (PEG) provides a steric barrier for liposomes in avoiding interactions with opsonins and the subsequent phagocytosis by the cells of the mononuclear phagocyte system (MPS) [1,2]. Consequently, PEGylated liposomes remain in circulation for a prolonged duration. PEGylated liposomes with a mean size of around 100 nm are attractive for tumor targeting because of a unique feature known as the enhanced permeability and retention (EPR) effect [3], for which the liposomes accumulate in tissue with leaky blood vessels (*i.e.*, tumors and inflamed tissue) after intravenous injection. The enlarged capillary gaps (>400 nm) in tumor vasculature allow penetration by PEGylated liposomes, which have prolonged circulating properties, from the blood into the interstitial spaces of tumor tissues. The most successful example of PEGylated liposomes in clinical use are the doxorubicin-containing PEGylated liposomes, known under the commercial name Doxil/Caelyx—that exploits polymer coating technology and the concept of the EPR effect.

However, it is well documented that an intravenous injection of PEGylated liposomes triggers rapid clearance of a subsequent dose of the same liposomes, when injected a few days later, in rats, mice and Rhesus monkeys [4-7]. This unusual and unexpected phenomenon is referred to as the accelerated blood clearance (ABC) phenomenon, indicating that empty PEGylated liposomes are immunogenic. On the basis of our earlier studies in rats, we proposed a tentative mechanism as follows: anti-PEG IgM, secreted in response to the first dose of PEGylated liposomes, selectively binds to the second dose of liposomes that is injected a few days later, and subsequently activates the complement systems. This, in turn, leads to opsonization of PEGylated liposomes with activated C3 fragments (iC3b) and to an enhanced uptake of the second dose of PEGylated liposomes by the Kupffer cells in the liver [8-10]. However, the details of the underlying mechanism for the induction of anti-PEG IgM production in the ABC phenomenon are yet to be elucidated. In addition, we have shown that the spleen plays an important role in the induction of the ABC phenomenon against PEGylated liposomes [8]. The spleen is known to be a highly organized secondary lymphoid organ that plays a central role in the primary defense against all types of antigens circulating in the bloodstream, and is a major site of antibody production [11,12]. It is assumed that the spleen produces anti-PEG IgM in the ABC phenomenon. However, direct evidence that the spleen secretes anti-PEG IgM in response to an injection of PEGylated liposomes has been scant.

Therefore, the present study was focused on the issue of whether anti-PEG IgM is produced with a similar mechanism in mice and rats and whether the spleen secretes anti-PEG IgM in mice and rats following intravenous injection of PEGylated liposomes. In particular, the questions asked were whether an anti-PEG IgM response against PEGylated liposomes occurs with a similar mechanism in mice and rats and whether splenic B-cells secrete anti-PEG IgM without the help of T-cells.

## 2. Materials and Methods

### 2.1. Animals

Male BALB/c mice, 4-5 weeks old, and Wistar male rats, 8 weeks old, were purchased from Japan SLC (Shizuoka, Japan). Male BALB/cAJcl-nu/nu mice and C.B-17/lcr-scid/scid mice, 4-5 weeks old, were purchased from Japan CLEA (Tokyo, Japan). The experimental animals were allowed free access to water and mouse chow, and were housed under controlled environmental conditions (constant temperature, humidity, and a 12 h dark-light cycle). All animal experiments were evaluated and approved by the Animal and Ethics Review Committee of the University of Tokushima.

### 2.2. Materials

Hydrogenated egg phosphatidylcholine (HEPC) and 1,2-distearoyl-sn-glycero-3-phosphoethanolamine-n-[methoxy (polyethylene glycol)-2000] (mPEG_2000_-DSPE) were generously donated by NOF (Tokyo, Japan). The cholesterol (CHOL) was of analytical grade (Wako Pure Chemical, Osaka, Japan). All lipids were used without further purification. All other reagents were of analytical grade.

### 2.3. Preparation of liposomes

PEGylated liposomes, composed of HEPC:CHOL:mPEG_2000_-DSPE (1.85:1:0.15, molar ratio), were prepared as in previous work [6]. Briefly, the lipids were dissolved in chloroform, and after evaporation of the organic solvent, the resulting lipid film was hydrated in HEPES buffered saline (25 mM HEPES, 140 mM NaCl, pH 7.4). The liposomes were sized by subsequent extrusion through polycarbonate membrane filters (Nucleopore, CA, U.S.) with pore sizes of 400, 200, 100, and 80 nm. The mean diameters of the prepared liposomes were determined using a NICOMP 370 HPL submicron particle analyzer (Particle Sizing System, CA, U.S.), and were 109.5 ± 29.8 nm. The concentration of phospholipid (PL) was determined by colorimetric assay [13].

### 2.4. Serum collection

Both mice and rats were intravenously injected with either PEGylated liposomes (1 μmol PL/kg and 0.001 μmol PL/kg, respectively) or HEPES buffered saline, except when studying the effect of dosage on anti-PEG IgM production. At day 5 after the injection of liposomes, mice and rats were euthanized and blood was withdrawn into tubes containing the serum separating agents (Eiken, Tokyo, Japan), which was allowed to stand for 30 min at room temperature. Serum was collected by centrifugation at 3,000 rpm and 4 °C for 15 min, then was frozen at -20 °C until further use.

### 2.5. Detection of anti-PEG IgM or IgG

A simple ELISA procedure, as described previously [14], was employed to detect anti-PEG IgM and anti-PEG IgG in the serum. Briefly, 10 nmol of mPEG_2000_-DSPE in 50 μL of 100% ethanol was added to a 96-well plate. To prepare lipid-coated plates, the plates were allowed to air dry completely for 2 h. Then, the plates were blocked for 1 h with Tris-buffered saline (50 mM Tris, 0.14 mM NaCl, pH 8.0) containing 1% BSA and subsequently were washed 3 times with Tris-buffered saline (pH 8.0) containing 0.05% Tween 20. Diluted serum samples (1:100) (100 μL) were then applied to the wells, followed by incubation for 1 h and 5 washes, as described above. Horseradish peroxidase (HRP)-conjugated antibody (100 μL, 1 μg/mL, Goat anti-mouse IgM IgG-HRP conjugate, Goat anti-mouse IgG IgG-HRP conjugate, or Goat anti-rat IgM IgG-HRP conjugate; Bethyl Laboratories, TX, U.S.) in the conjugate diluents (Tris-buffered saline (pH 8.0) containing 1% BSA and 0.05% Tween 20) was added to each well. After 1 h incubation, the wells were washed five times with a wash solution, as described above. Coloration was initiated by the addition of 100 μL of o-phenylene diamine (1 mg/mL) (Sigma, MO, U.S.). After an appropriate incubation time (5–10 min), the reaction was stopped by adding 100 μL of 2 N H_2_SO_4_, and the absorbance was measured at 490 nm using a Microplate reader (Sunrise, TECAN Japan, Kanagawa, Japan). All incubations were performed at room temperature.

### 2.6. Surgical removal of the spleen (splenectomy)

Splenectomy was performed on mice and rats anesthetized intraperitoneally with pentobarbital sodium (50 mg/kg) 1 day before PEGylated liposomes were given [8]. An incision was made in the left flank, and the spleen was isolated and removed using a cautery instrument. This procedure ensured that the spleen was removed in total and that no splenic fragments were left behind, which was confirmed by examination of the mice at the time of euthanasia.

### 2.7. Statistics

All mean values are expressed as the mean ± S.D. Statistical analysis was performed with a two-tailed unpaired Student's t-test using GraphPad InStat software (GraphPad Software, CA, U.S.). The level of significance was set at *p* < 0.05.

## 3. Results and Discussion

### 3.1. Induction of anti-PEG IgM production in mice

**Figure 1.**
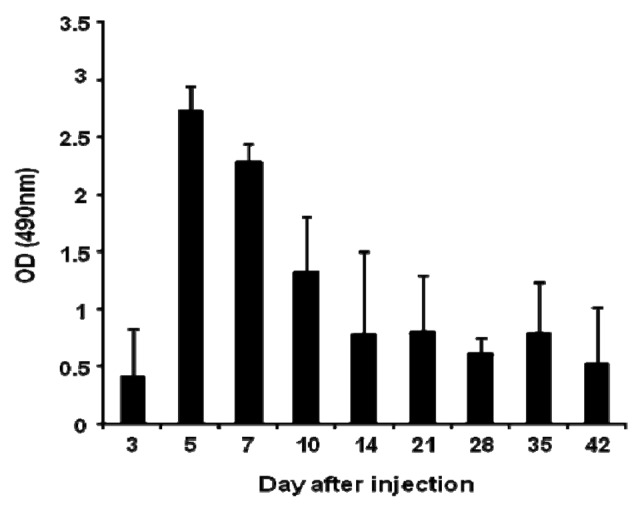
Anti-PEG IgM production following a single intravenous injection of PEGylated liposomes in mice. PEGylated liposomes were intravenously injected into mice at a dose of 1 μmol PL/kg. Sera were collected on days 3, 5, 7, 10, 14, 21, 28, 35, and 42 after injection. Anti-PEG IgM was detected using ELISA, as described in the Material and Methods section. Each value represents the mean ± S.D. (n = 4).

Anti-PEG IgM production was detected in mice following a single intravenous injection of PEGylated liposomes at a dose of 1 μmol PL/kg (Figure 1). The level of anti-PEG IgM in serum began to increase at day 3, peaked at day 5, and then gradually decreased. This finding is consistent with earlier results using rats [14].

**Figure 2.**
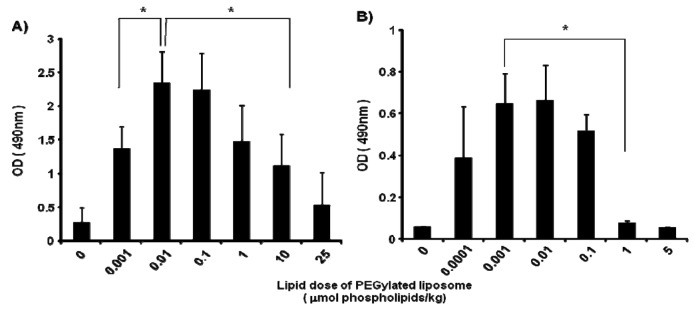
Effect of lipid dose of PEGylated liposomes on anti-PEG IgM production in (A) mice and (B) rats. Mice were injected with PEGylated liposomes at doses of 0, 0.001, 0.01, 0.1, 1, 10, or 25 μmol PL/kg. Rats were injected with liposomes at doses of 0, 0.0001, 0.001, 0.01, 0.1, 1, or 5 μmol PL/kg. Serum was collected on day 5 after injection. Anti-PEG IgM in the serum was determined using ELISA, as described in the Material and Methods section. Each value represents the mean ± S.D. (n = 4). *p < 0.05.

In mice, the anti-PEG IgM production at day 5 after injection was affected by the lipid dose of PEGylated liposomes (0, 0.001, 0.01, 0.1, 1, 10 and 25 μmol PL/kg) (Figure 2A). The level of anti-PEG IgM production was increased as the lipid dose was increased to 0.01 μmol PL/kg and decreased as the lipid dose was increased further. A similar tendency for anti-PEG IgM production was observed in rats (Figure 2B); the level increased as the lipid dose increased to 0.001 μmol PL/kg and decreased with further increases in the lipid dose. The rats appeared to be somewhat more sensitive than the mice to the PEGylated liposomes.

### 3.2. Effect of splenectomy on anti-PEG IgM production

**Figure 3.**
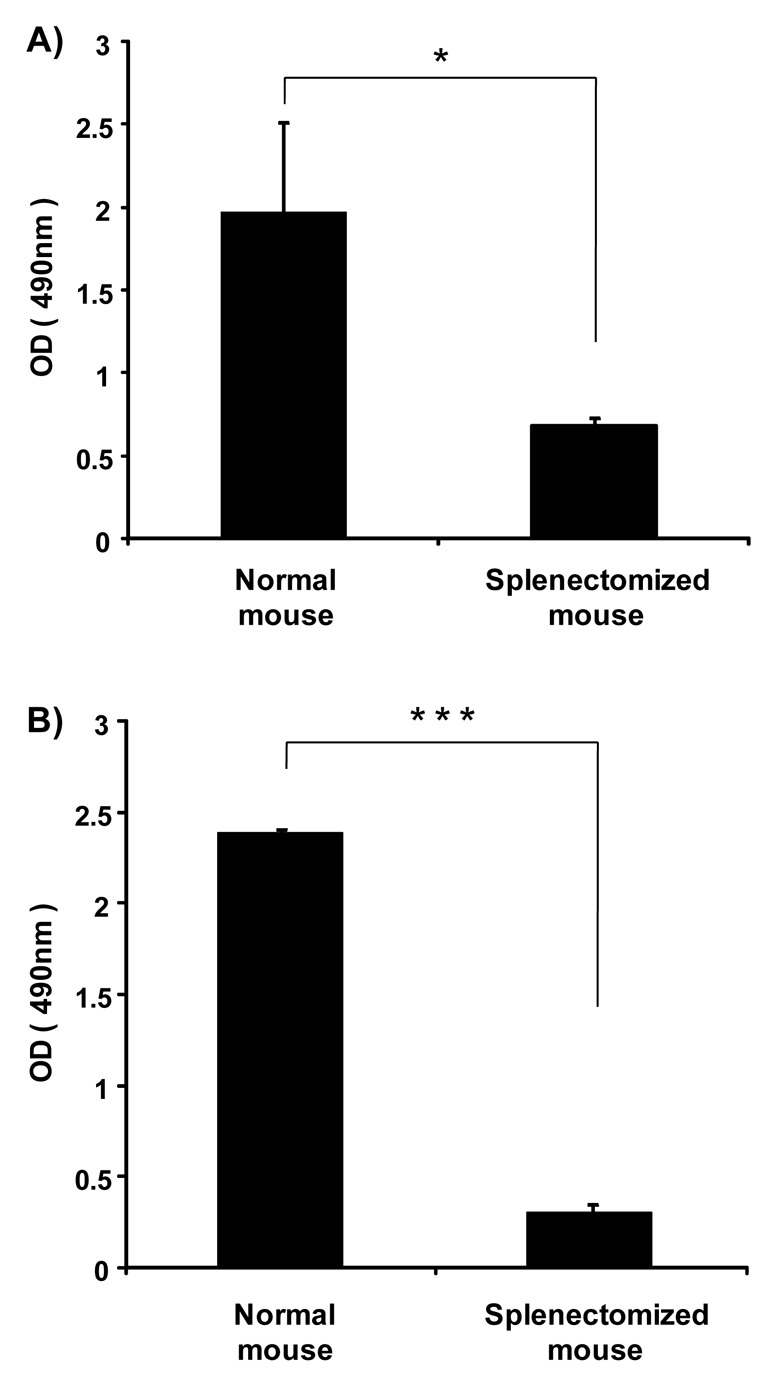
Effect of splenectomy on anti-PEG IgM production in (A) mice and (B) rats. The animals were divided into two groups: splenectomized and non-splenectomized. The former received splenectomy one day before the injection of PEGylated liposomes; the latter were not splenectomized. The mice were injected with PEGylated liposomes at a dose of 1 μmol PL/kg. The rats were injected with PEGylated liposomes at a dose of 0.001 μmol PL/kg. At day 5 after the injection, serum was collected and anti-PEG IgM was determined using ELISA, as described in the Material and Methods section. Each value represents the mean ± S.D. (n = 4). *p < 0.05, *** *p* < 0.005.

Previously, we showed that the spleen plays an important role in the induction of the ABC phenomenon in rats [8]. However, there was no direct evidence to indicate that the spleen contributes to the production of anti-PEG IgM in mice and rats. Here, we investigated the effect of splenectomy (removal of the spleen) on anti-PEG IgM production in mice and rats. Anti-PEG IgM production was obvious in normal mice that were used as the positive control, while the production was significantly reduced in splenectomized mice (Figure 3A). A similar reduction of anti-PEG IgM was observed in splenectomized rats, compared to normal rats (Figure 3B). These results suggest that the spleen is a major organ involved in the secretion of anti-PEG IgM in mice and rats.

### 3.3. Anti-PEG IgM response in immunodeficient mice

**Figure 4.**
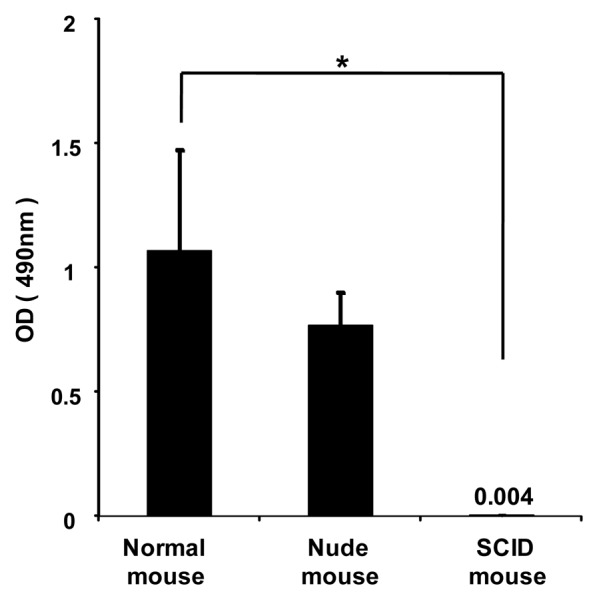
Anti-PEG IgM production following a single intravenous injection of PEGylated liposomes in immunodeficient (nude and SCID) mice. The mice were injected with PEGylated liposomes at a dose of 1 μmol PL/kg. At day 5 after the injection, serum was collected and anti-PEG IgM was determined with ELISA as described in the Material and Methods section. Each value represents the mean ± S.D. (n = 3). *p < 0.05.

To investigate whether anti-PEG IgM is secreted from B-cells without the help of T-cells, PEGylated liposomes were intravenously injected into immunodeficient athymic (nude) mice that were B-cell deficient and severe combined immunodeficient (SCID) mice that were B- and T-cell deficient. In the normal mice, PEGylated liposomes caused the production of anti-PEG IgM (Figure 4). The nude mice experienced anti-PEG IgM production that was similar to that of normal mice, while the SCID mice experienced no production. This suggests that B-cells secrete anti-PEG IgM without the help of T-cells following the injection of PEGylated liposomes.

### 3.4. Effect of repeated injections of PEGylated liposomes on anti-PEG IgM production in mice

**Figure 5.**
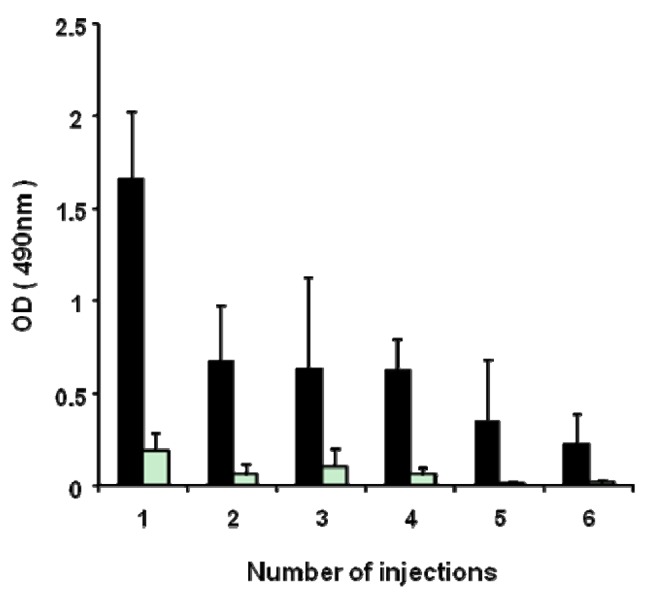
Anti-PEG IgM production following sequential injections with PEGylated liposomes in mice. PEGylated liposomes (1 μmol PL/kg) were intravenously injected six times in 7-day intervals in mice. Serum was collected at day 5 after each injection. Anti-PEG IgM and anti-PEG IgG in the serum was determined with ELISA as described in the Material and Methods section. The closed column represents anti-PEG IgM, and the open column represents anti-PEG IgG. Each value represents the mean ± S.D. (n = 4).

As shown in Figure 5, the anti-PEG IgM response against PEGylated liposomes occurred in a T-cell-independent manner. PEGylated liposomes seem to act as a T-cell-independent (TI) antigen in anti-PEG IgM. It is generally known that the TI antigen has poor immunological memory and induces lesser immunological class switching from IgM to IgG [15]. Accordingly, it was expected that repeated injections of PEGylated liposomes would not boost anti-PEG IgM response and cause immunological class switching from IgM to IgG. In the present study, PEGylated liposomes (1 μmol PL/kg) were repeatedly injected six-times into the same mice at seven-day intervals. A serum sample was obtained from the mice on day 5 after each injection. The first dose induced a maximum level of anti-PEG IgM production (Figure 5). After priming with sequential doses, the anti-PEG IgM response was not boosted. Throughout the treatments, few anti-PEG IgG responses were induced (Figure 5). It appeared that the anti-PEG response against PEGylated liposomes did not include isotype switching from IgM to IgG and did not generate B-cell memory.

### 3.5. Discussion

The ABC phenomenon is a crucial issue in the development of novel colloidal nanocarriers, because their pharmacokinetics after intravenous injection must be reproducible in a clinical setting to prevent unanticipated side effects and preserve pharmacological activity. To avoid this phenomenon, technological improvements in the design of PEGylated nanocarriers and elucidation of the mechanism of the phenomenon are both very important. In earlier studies, the first dose of empty PEGylated liposomes elicited anti-PEG IgM secretion, which was responsible for rapid clearance of the second dose of PEGylated liposomes injected a few days later in the same animals [16]. In addition, the spleen played an important role in the induction of the ABC phenomenon [8]. In the present study, we could show that splenic B-cells produce anti-PEG IgM in response to an injection of PEGylated liposomes (Figure 3 and 4).

Although antibody responses to most protein antigens are dependent on helper T-cells, humans and mice with T-cell deficiencies nevertheless make antibodies against many bacterial antigens [15]. Such bacterial antigens are known as T-cell-independent (TI) antigens. These TI antigens induce only limited isotype switching from IgM to IgG and do not induce memory B-cells that produce IgG antibodies. In the present study, anti-PEG IgM production was observed in T-cell deficient nude mice, but not in B- and T-cell-deficient SCID mice (Figure 4), suggesting that the anti-PEG IgM response against PEGylated liposomes occurred without T-cell help. In addition, serial injections with PEGylated liposomes did not promote isotype switching and anti-PEG IgG production (Figure 5). Thus, it was confirmed that PEGylated liposomes must be classified as T-cell independent (TI) (or thymus independent antigen), as reported previously [14,17]. The TI antigens generally fall into two classes (TI-1 or TI-2), which activate B-cells by two different mechanisms. TI-1 antigens, such as bacterial lipopolysaccharides, are potent B-cell (mature and immature) mitogens, capable of polyclonal IgM production [18,19]. By contrast, TI-2 antigens only activate mature B-cells and consist of highly repetitive structures, such as capsular polysaccharide [18,19]. Liposomal PEG has a repeating O-CH_2_-CH_2_ subunit in the structure, which is typical of TI-2 antigens consisting of highly repetitive structures, such as capsular polysaccharide [15]. Repeating polymers such as polyvinylpyrrolidone (PVP) and conventional liposomes without PEG modification were shown as examples of TI-2 antigens in an earlier study [20]. Although precise studies are required, it can be assumed that PEGylated liposomes may function as a TI-2 antigen in an anti-PEG IgM response.

In the present study, anti-PEG IgM production was transiently increased in mice following a single injection of PEGylated liposomes (Figure 1), which is consistent with an earlier report involving rats [14]. Splenectomy remarkably reduced the anti-PEG IgM response in both mice and rats (Figure 3). The anti-PEG IgM production response to an injected lipid dose was very similar in both mice and rats (Figure 2). Thus, the conclusion was that anti-PEG IgM responses against PEGylated liposomes occur in a similar mechanism to that in rats, and since it is confirmed that the ABC phenomenon relates to an anti-PEG IgM response against PEGylated liposomes, immunological investigations are necessary to further elucidate the underlying mechanism. Immunological techniques and antibodies to the cellular differentiation (CD) markers of lymphocytes are more available for mice than for rats. The animals targeted for experimentation will be switched from rats to mice in order to accelerate the progress of the research related to the ABC phenomenon.

It is noteworthy that in the response to an injected lipid dose, rats were somewhat more sensitive than mice to PEGylated liposomes, although the anti-PEG IgM was finally produced in a similar fashion (Figure 2). This might be due to the difference in pharmacokinetics of PEGylated liposomes between rats and mice. Accumulation of PEGylated liposomes in the spleen was much larger in rats than in mice [6,7]. A larger accumulation of PEGylated liposomes in the spleen might provide stronger stimulation to the immune systems of mice.

Thus far, considerable efforts have been made to develop various PEGylated carriers such as liposomes, nano-size particles and polymeric micelles [21,22]. The induction of the ABC phenomenon has been reported in studies on other PEGylated carriers such as polymeric micelles [23,24] and solid nanoparticles [25]. The ABC phenomenon that leads to unusual pharmacokinetics for a PEGylated carrier system upon repeated injections would block the expansion of future clinical applications. Although the underlying mechanism for induction of the anti-PEG IgM response against PEGylated liposomes is not clear yet, the results of the present study should help with the design of a PEGylated nanocarrier system that would be capable of avoiding, or weakening, the ABC phenomenon.

## 4. Conclusion

The anti-PEG IgM production seems to occur with a similar mechanism in mice and rats. The spleen proved to be a major organ in the secretion of anti-PEG IgM in mice and rats. Anti-PEG IgM production was detected in nude, T-cell deficient mice, but not in SCID mice with B- and T-cell deficiencies. In addition, sequential injections of PEGylated liposomes into the same mice did not promote isotype switching from IgM to IgG. Accordingly, PEGylated liposomes may be categorized as TI-2 antigens. Although the underlying mechanism for the inducement of an anti-PEG IgM response against PEGylated liposomes is not yet clear, the results of the present study should help elucidate the anti-PEG IgM response against PEGylated liposomes.

## Abbreviations

ABC: accelerated blood clearance
CD: cellular differentiation
CHOL: cholesterol
EPR: enhanced permeability and retention
HEPC: hydrogenated egg phosphatidylcholine
HRP: horseradish-peroxidase
mPEG_2000_-DSPE: 1,2-distearoyl-sn-glycero-3-phosphoethanolamine-n-[methoxy (polyethylene glycol)-2000]
MPS: mononuclear phagocyte system
PEG: polyethyleneglycol
PL: phospholipid
SCID: severe combined immunodeficient
TI antigen: T-cell independent antigen
